# Les complications de l’ECMES dans le traitement des fractures des 2 os de l’avant-bras chez l’enfant (à propos de 87 cas)

**DOI:** 10.11604/pamj.2017.27.68.11058

**Published:** 2017-05-30

**Authors:** Saad Andaloussi, Mohamed Amine Oukhouya, Othmane Alaoui, Karima Atarraf, Lamiae Chater, My Abderrahmane Afifi

**Affiliations:** 1 Service de Traumato-Orthopédie Pédiatrique, CHU Hassan II, Fès, Maroc

**Keywords:** Fracture, avant-bras, embrochage centromédullaire élastique stable, enfant, Fracture, forearm, elastic stable intramedullary nailing, child

## Abstract

Le but de notre étude est de décrire les complications de l'embrochage centromédullaire élastique stable (ECMES) dans le traitement des fractures diaphysaires des deux os de l'avant-bras chez l'enfant. Entre janvier 2009 et décembre 2013, 87 enfants présentant des fractures diaphysaires des os de l'avant-bras ont été traités par embrochage centro-médullaire élastique stable par des broches de Métaizeau. Il s'agissait de 76 garçons et 11 filles d'âge moyen 12 ans. L'embrochage a été réalisé d'emblée dans 50 cas et suite à un déplacement secondaire sous plâtre dans les autres. Les deux os ont été embrochés dans tous les cas. L'immobilisation plâtrée était systématique pour tous les malades pendant une durée moyenne d'un mois. Les broches ont été enlevées au bout d'environ 6 mois en moyenne. Les résultats fonctionnels ont été étudiés avec un recul moyen de 10 mois. Les complications ont été marquées par 14 infections superficielles, 2 cas d'ostéites sur matériel, 3 cas de refracture, 3 cas de pseudarthrose, 2 cas de retard de consolidation et une synostose radio-cubitale proximale. Bien que l'embrochage centro-médullaire soit une méthode d'ostéosynthèse idéalement adaptée aux enfants, il reste plus invasif que le traitement orthopédique. Ses indications doivent rester dans les limites de celui ci.

## Abstract (en)

This study aims to describe the complications of elastic stable intramedullary nailing (ESIN) in the treatment of both-bone forearm diaphyseal fractures in the child. Between January 2009 and December 2013, 87 children with both-bone forearm diaphyseal fractures were treated by elastic stable intramedullary nailing with Métaizeau nails. 76 boys and 11 girls, with an average age of 12 years, were enrolled in the study. Nailing was promptly performed in 50 cases and after secondary displacement during plaster-cast treatment in the other cases. Both bones were nailed in all cases. All patients underwent systematic plaster immobilization for a period of about one month. On average, nails were removed after about 6 months. Functional outcomes were studied over a mean follow-up period of 10 months. Complications were marked by 14 superficial infections (14 cases), osteitis associated with material (2 cases), refracture (3 cases), pseudarthrosis (3 cases), delayed fracture consolidation (2 cases) and proximal radioulnar synostosis (1 case). Although intramedullary nailing ideally is an osteosynthesis technique suitable for the treatment of fractures in children, it is more invasive than orthopaedic treatment.Indications for treatment should remain within well-established limits.

## Introduction

L'ECMES ou l'embrochage centro-médullaire élastique stable est devenu le traitement chirurgical de référence des fractures de l'avant-bras chez l'enfant et l'adolescent. Sur le plan biologique, l'ECMES reprend tous les avantages du traitement orthopédique : respect de l'hématome fracturaire, du périoste et du cartilage de croissance, conservation de la micromobilité tout en supprimant la macromobilité. En outre, il supplée aux carences de l'immobilisation plâtrée sur le plan fonctionnel: l'ECMES assure la stabilité du foyer de fracture, il réduit le risque de cal vicieux et de syndrome compartimental. Sur le plan social, l'autonomie de l'enfant est respectée grâce à l'absence d'immobilisation prolongée [1, 2]. L'ECMES n'offre toutes ces qualités que lorsqu'il est réalisé dans les règles de l'art: ce n'est pas un embrochage d'alignement. C'est le cintrage des broches et le positionnement précis de celles-ci qui leur confèrent toute leur efficacité. Il est important de se rappeler que chez l'enfant le périoste joue un rôle fondamental dans la consolidation fracturaire. Au titre des désavantages et comparé au traitement orthopédique, l'ECMES reste toutefois un traitement chirurgical avec ses inconvénients propres. Le temps d'irradiation en radioscopie est plus long, le risque infectieux est présent, les cicatrices, bien que réduites, existent et l'ablation du matériel doit être envisagée. Cette étude se propose de montrer les différentes complications de l'embrochage centro-médullaire élastique stable des fractures de l'avant-bras.

## Méthodes

Nous avons revu pour ce travail, 87 observations d'embrochage centro-médullaire élastique stable des deux os de l'avant bras chez l'enfant colligées au service de Traumato-Orthopédie pédiatrique du CHU Hassan II de Fès sur une période de 5 ans allant de janvier 2009 jusqu'au décembre 2013. Les résultats fonctionnels ont été étudiés avec un recul moyen de 10 mois et des extrêmes allant de 6 mois à 4 ans. Notre série a comporté 76 garçons (87%) et 11 filles (13%), d'âge moyen 11,1 ans avec des extrêmes de 5 et 16 ans. L'atteinte du côté gauche était prédominante avec 44 cas (54%). Le mécanisme était indirect dans la totalité des cas. Les deux os ont été fracturés ensemble dans tous les cas. Le siège des fractures se situait principalement au niveau des deux tiers inférieurs. Les lésions cutanées étaient les complications traumatiques les plus fréquentes (27%). La fracture était ouverte dans 24 cas, dont 17 classés stade I de Cauchoix et Duparck, 7 classées stade II. Dans notre série, le caractère ouvert n'était pas une indication à un traitement chirurgical d'emblée même si celui-ci était préféré en raison de la facilité de surveillance de la plaie dans les suites opératoires. Un syndrome de Wolkman a été noté dans un cas. Le traitement orthopédique a été tenté, de première intention, dans la majorité des cas. Le recours à l'embrochage centro-médullaire élastique stable n'a été fait qu'après échec des tentatives du traitement orthopédique. Les indications ont intéressé les fractures irréductibles (72 cas), les fractures instables (14 cas), le syndrome de Wolkman (1 cas). La réduction à ciel ouvert sur l'un des deux os au moins a été nécessaire dans 32 cas (36%). L´abord du foyer de fracture permet de dégager le muscle incarcéré et d´aligner directement les deux fragments. L'embrochage centro-médullaire a été fait pour tous les malades par une broche de Métaizeau de 2 à 2,5 mm. Une immobilisation plâtrée par une attelle brachio-antébrachio-palmaire postérieure à visée antalgique, coude fléchi à 90°, de règle chez tous les malades, a été maintenue pour une durée moyenne de 4 semaines. L'ablation des broches a été réalisée au bout de 6 mois en moyenne sous anesthésie générale après contrôle radiologique de la consolidation osseuse.

## Résultats

**Figure 1:**
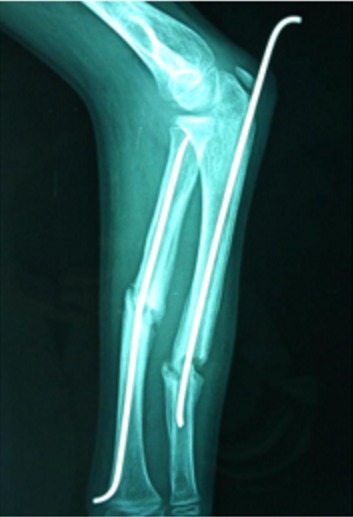
Migration de la broche cubitale

**Figure 2:**
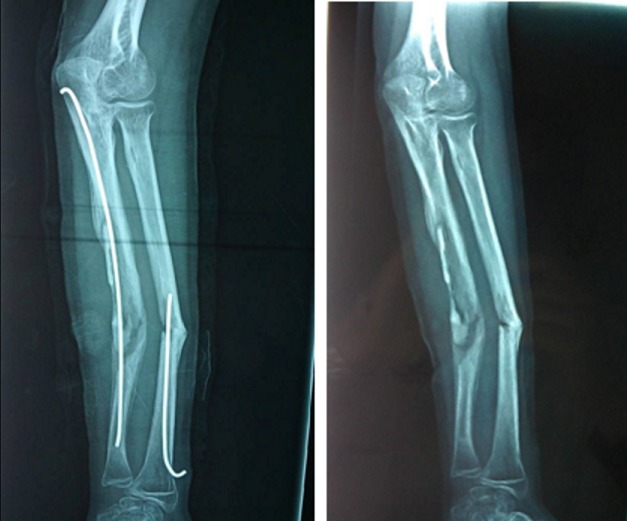
Ostéite

**Figure 3:**
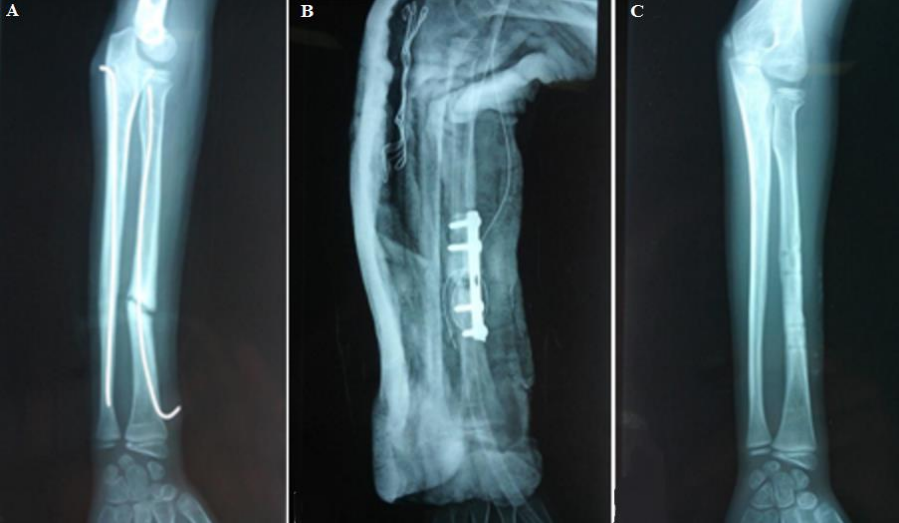
(A) Pseudarthrose du radius; (B) ostéosynthèse par plaque; (C) radio après 8 mois

**Figure 4:**
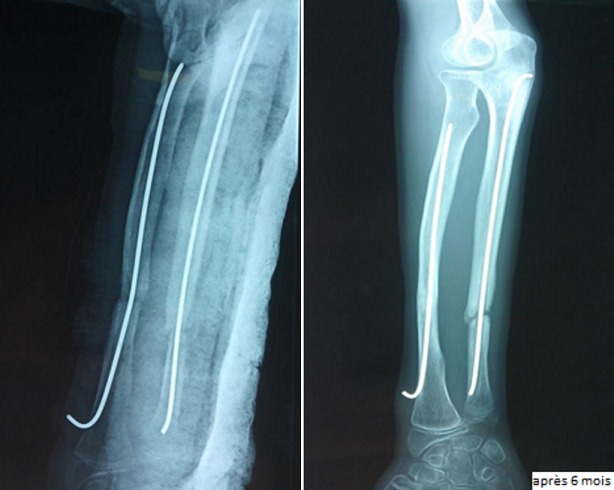
Retard de consolidation du cubitus après 6 mois

Parmi les 87 cas, la réduction était anatomique avec une consolidation dans les délais chez 76 malades (89%). Aucune complication postopératoire précoce nerveuse ou tendineuse n´a été signalée. Les complications secondaires ont été dominées par l´irritation cutanée par la pointe de la broche. Ainsi, nous avons enregistré 14 infections superficielles (17%). Ces infections ont été traitées par une antibiothérapie orale et des soins locaux. Dans un cas, il s'agissait d'une migration de la broche cubitale qui a été recoupée (Figure 1). Nous avons noté 2 cas d´ostéite sur matériel pour des fractures ouvertes classées classe II de Cauchoix et Duparck (Figure 2). Les complications tardives ont été marquées par 3 cas de refracture sur matériel (3,5%), à cause d´une nouvelle chute, ayant bénéficié d'une réduction sur broches en place. Nous avons noté 3 cas de pseudarthrose (3,5%), sur des fractures dont le foyer a été abordé chirurgicalement, nécessitant un avivement des berges avec ostéosynthèse par plaque (Figure 3). Deux cas (2,3%) de retard de consolidation (ayant consolidés après 9 et 11 mois) ont été remarqués et ils ont nécessité de prolonger la durée de l´ostéosynthèse (Figure 4). Une synostose radio-cubitale proximale est survenue dans un cas. La plupart des fractures (94%) ont consolidé dans un délai moyen de 8 semaines avec des extrêmes allant de 6 à 14 semaines. Nous n'avons pas noté de différence significative dans ce délai en fonction de l'âge, du siège de la fracture, du type de la fracture, du type de la réduction et des complications cutanées.

## Discussion

S'il est admis que bon nombre d'imperfections de réduction peuvent aboutir à une restitution anatomique et fonctionnelle par effet remodelant de la croissance, cette correction a des limites et certains cals vicieux peuvent rester définitifs et se solder par des séquelles fonctionnelles [3–5]. L'embrochage centro-médullaire élastique stable présente des avantages réels sur les autres techniques chirurgicales. Il permet le respect de l'hématome fracturaire et du périoste surtout s'il est réalisé à foyer fermé [3]. Comme toute technique chirurgicale, l'embrochage centro-médullaire élastique stable n'est pas dénué de complications. Mc Cullen [6] a rapporté un taux de complications de 55%, mais rares sont celles qui affectant le résultat fonctionnel final. Les principales complications sont : la migration des broches, les infections, les lésions nerveuses, la synostose radio-cubitale, les retards de consolidation, les ruptures tendineuses et les déplacements secondaires [7, 8]. Prevot [9] a rapporté dans sa série de 125 factures, 2 lésions tendineuses, 3 cas d'hypoesthésie transitoire, 11 cas d'irritation cutanée par les broches, 2 cas de torsion de la broche, un cas de cassure de la broche, un cas de retard de consolidation et 5 fractures itératives. Les infections superficielles au niveau des orifices des broches sont assez fréquentes mais sont de bon pronostic si elles sont traitées précocement. L'enfouissement des broches aidera à diminuer leur incidence. Mais le plus souvent, l'extrémité des clous est naturellement saillante pour pouvoir permettre leur ablation. Rappelons que l'ablation de l'ECMES est une intervention chirurgicale non dénuée de risque [10]. Il faut utiliser une pince coupante qui coupe les clous de façon nette et non tranchante. Puis, à l'aide d'un impacteur, il faut repousser les clous de façon à ne laisser en dehors de l'os que 3 à 5 mm. La protection des extrémités métalliques par un morceau de drain de caoutchouc ou un capuchon en polyéthylène améliore la tolérance des parties molles au contact du métal. Quant aux ostéites, elles restent rares après ECMES, de l'ordre de 0,2% [11], mais leur risque persiste malgré les meilleures actions préventives menées et elles font perdre à l'embrochage centro-médullaire son innocuité. Les pseudarthroses sont rarement rapportées en dehors de fractures survenant dans un contexte de sévérité du traumatisme initial très violent. Quant aux retards de consolidation, ils ne sont considérés qu'au-delà de 3 mois, délai normal pour ces fractures diaphysaires. Certaines conditions peuvent retarder l´apparition du cal principalement lorsque les conditions bio-mécaniques du montage ne sont pas optimales: les broches sont trop fines autorisant une mobilité trop importante dans le foyer ou au contraire les broches sont trop grosses inhibant le cal périosté en réalisant une immobilisation trop stricte. L'ouverture d'un foyer de fracture, qu'il soit d'origine, comme dans une fracture ouverte, ou lié à un abord chirurgical lors d'un ECMES, est responsable d'un allongement du délai de consolidation radiologique [12]. La principale difficulté dans la réalisation de l'ECMES est le passage du foyer de fracture et la cathétérisation du fragment osseux opposé, ce d'autant plus que la réduction obtenue préalablement est imparfaite, sans alignement des fragments. La broche se perd alors dans les tissus mous, risquant de léser des éléments nobles comme cela a été décrit par Lidder et al qui rapporte une lésion du nerf médian au décours d'une tentative d'embrochage [13], voire de majorer l'œdème dans les loges musculaires et favoriser la survenue d'un syndrome compartimental [14]. Certains estiment même qu'une mini incision en regard du foyer de fracture afin de réduire et de permettre l'embrochage, cause un traumatisme tissulaire beaucoup moins important que de multiples tentatives de réduction par manœuvres externes [15]. Parallèlement, chaque tentative de cathétérisation du fragment opposé s'accompagne de nouveaux contrôles scopiques, ce qui peut amener à une importante dose cumulée de rayonnement en fin d'intervention, qui s'ajoutera aux différents clichés radiologiques nécessaires pour le diagnostic puis le suivi de la consolidation. En ce sens, certains auteurs rappellent que le tissu osseux de l'enfant est un tissu prolifératif, par rapport à celui de l'adulte, et par conséquent beaucoup plus sensible à l'effet nocif des rayonnements ionisants [16, 17]. A l'opposer, un abord systématique du foyer de fracture n'est pas une attitude à retenir aux vues des inconvénients et complications rapportés après abord. En effet, plusieurs études rapportent une corrélation entre l'abord du foyer de fracture et un risque accru de retard de consolidation par suppression de l'hématome au niveau du foyer de fracture [18, 19] et des facteurs ostéo-inducteurs endogènes contenus dans celui-ci. Ainsi, Fernandez et al a confirmé un taux de pseudarthrose plus important en cas de fracture ouverte ou d'abord du foyer [20] alors que Flynn et al chiffre à deux semaines l'augmentation du délai de consolidation en cas d'abord du foyer de fracture [21]. Dans notre série, également, les cas de pseudarthrose et de retard de consolidation sont survenus après abord du foyer de fracture. Au total, le chirurgien est donc partagé entre deux contraintes opposées, à savoir, d'une part ne pas augmenter de façon déraisonnable le nombre de tentatives en cas d'échec. D'autre part, ne pas succomber à la facilité d'un abord systématique du foyer de fracture, d'où l'intérêt d'isoler des facteurs prédictifs d'échec permettant dans ce cas uniquement de décider précocement d'un abord du foyer de fracture. Enfin, le taux d'abord chirurgical retrouvé dans notre étude était de 36%, se situant dans la moyenne des taux rapportés dans la littérature (12 à 52%) [22– 25].

## Conclusion

Tout en admettant que l'embrochage centro-médullaire soit une procédure peu invasive, il reste plus invasif que le traitement orthopédique et doit par conséquent n'être recommandé que si le résultat fonctionnel optimal ne peut être obtenu par réduction fermée et immobilisation plâtrée. Si sa technique de réalisation est parfaitement respectée, l´ECMES est un moyen d´ostéosynthèse fiable n´exposant le plus souvent qu´à des complications mineures, qu´il est facile d´éviter ou de contrôler.

### Etat des connaissances actuelle sur le sujet

- les principes techniques de réalisation de l'embrochage centro-médullaire élastique stable;
- les avantages et les inconvénients de cette ostéosynthèse.

### Contribution de notre étude à la connaissance

- exposer l'expérience du service de traumato-orthopédie pédiatrique du CHU Hassan II de Fès en matière de fractures des 2 os de l'avant-bras traitées par ECMES;
- évaluer les complications postopératoires de cette technique sur une large série.

## Conflits d’intérêts

Les auteurs ne déclarent aucun conflit d'intérêt.
